# Genetic Variants of *Orientia tsutsugamushi* in Domestic Rodents, Northern China

**DOI:** 10.3201/eid1907.120984

**Published:** 2013-07

**Authors:** Meng Zhang, Zhong-Tang Zhao, Xian-Jun Wang, Zhong Li, Lei Ding, Shu-Jun Ding, Hui-Li Yang

**Affiliations:** Shandong University, Jinan, People’s Republic of China (M. Zhang, Z.-T. Zhao, L. Ding);; Shandong Center for Disease Control and Prevention, Jinan (X.-J. Wang, Z. Li, S.-J. Ding);; Tai’an Center for Disease Control and Prevention, Tai’an, China (H.-L. Yang)

**Keywords:** scrub typhus, *Orientia tsutsugamushi*, genotype, phylogeny, parasites, China, rodents, humans

## Abstract

We screened *Orientia tsutsugamushi* from 385 domestic rodents and 19 humans with scrub typhus in rural Tai’an District, Shandong Province, a new scrub typhus epidemic area in northern China. Sequence analysis identified 7 genotypes in the rodents, of which 2 were also identified in the humans.

*Orientia tsutsugamushi*, the causative agent of scrub typhus, is widely prevalent in the Asia–Pacific region and causes an estimated 1 million cases per year (*1*). It is characterized by dramatic genetic diversity (*2*). In the 1960s, complement fixation initially identified *O. tsutsugamushi* as Karp, Gilliam, and Kato types (*3*), which are widely used as tested antigens in serologic assays. Since then, >20 antigenic variants of *O. tsutsugamushi* have been identified by immunologic and molecular methods (*2*). The 56-kDa type-specific antigen (TSA), which is one of the major immunogens of the agent and is associated with pathogenesis, has been commonly used for type designation (*4*–*6*).

Scrub typhus is a traditional tropical rickettsiosis. However, since 1986, it has emerged and spread rapidly in temperate zones; the epidemic season is mainly in autumn and winter (*7*,*8*). In the late 1990s, the district of Tai’an, west of Shandong Province, northern China (116°20′–117°59′E, 35°38′–36°28′N), became a new epidemic area of autumn–winter type scrub typhus (*9*); however, few epidemiologic and molecular investigations of the transmission cycle of the disease have been conducted in this area. We therefore investigated the prevalence of *O. tsutsugamushi* infection among rural domestic rodents in the newly developed epidemic area, evaluated the genotypic diversity according to the variations in the 56-kDa TSA gene, and explored the genetic relationship between strains circulating among domestic rodents and those infecting humans.

## The Study

From September 2010 through March 2012, rodents were trapped in rural residences from 2 county-level divisions (Xintai and Daiyue) in Tai’an. After each rodent was numbered and species identified, the spleen was dissected for DNA extraction.

During 2010–2011, scrub typhus patients from Xintai and Daiyue were recruited from 7 hospitals in Tai’an. Before the patients received antimicrobial drugs, whole blood samples were collected and anticoagulated with EDTA-Na_2_. Eschar specimens, if available, were collected after spontaneous desquamation.

DNA was extracted from each specimen by using a TIANamp Genomic DNA Kit (TIANGEN, Beijing, China). Considering the higher sensitivity and specificity, seminested PCR was used to amplify the coding sequences spanning variable domain I–VD III of the 56-kDa TSA with previously described primers A (5′-TTTCGAACGTGTCTTTAAGC-3′), B (5′-ACAGATGCACTATTAGGCAA-3′), and E (5′-GTTGGAGGAATGATTACTGG-3′) (*10*), which yielded fragments of ≈733 nt. To avoid contamination, PCR preparation was performed in an area separate from DNA extraction, and negative controls were included in each step. Nucleotide sequences of *O. tsutsugamushi* determined in this study were deposited in GenBank under accession nos. JX202566–JX202568, JX202573, JX202578, JX202579, and JX202580–JX202589. Theses sequences were aligned with 38 reference sequences of the *O. tsutsugamushi* 56k-Da TSA gene retrieved from GenBank by using MEGA software with the ClustalW algorithm (*11*). The phylogenetic tree was constructed by using MEGA software with the minimum-evolution method and the Kimura 2-parameter model, with bootstrapping for 1,000 replications. A percentage nucleotide identity matrix was calculated by using MegAlign (Lasergene; DNASTAR Inc., Madison, WI, USA).

The species of the 385 captured rodents were 244 *Rattus norvegicus*, 139 *Mus musculus*, and 2 *R. rattus*. *O. tsutsugamushi* DNA was detected by PCR in 10 (2.6%) rodents captured in October, November, December, and March; 6 (4.3%) rodents were *M. musculus* and 4 (1.6%) were *R. norvegicus*. Rates of *O. tsutsugamushi* positivity in domestic rodents captured in spring, summer, autumn, and winter were 5.3%, 0, 3.1%, and 1.2%, respectively (Table). No significant difference was observed in the rate of *O. tsutsugamushi* positivity determined by PCR among the 3 species of rodents (p = 0.278) or among seasons (p = 0.274).

**Table:** Rates of *Orientia tsutsugamushi* positivity among domestic rodents , Tai’an, China, September 2010–March 2012*

Rodent species	Season, no. positive/no. tested (%)				
	Spring	Summer	Autumn	Winter	Total
*Mus musculus*	2/18 (11.1)	0/21 (0)	3/60 (5.0)	1/40 (2.5)	6/139 (4.3)
*Rattus norvegicus*	1/39 (2.6)	0/31 (0)	3/131 (2.3)	0/43 (0)	4/244 (1.6)
*R. rattus*	0	0	0	0/2 (0)	0/2 (0)
Total	3/57 (5.3)	0/52 (0)	6/191 (3.1)	1/85 (1.2)	10/385 (2.6)
*Determined by PCR.					

Among the 10 sequences obtained from rodents, we identified 3 genogroups (Kawasaki-related, Fuji-related, and novel genogroup) involving 7 genotypes with >1 nt difference. The genotypes showed 69.7%–99.9% identity with each other. *M. musculus* and *R. norvegicus* rodents in Tai’an could serve as reservoirs of 5 and 3 genotypes, respectively. Genotypes KWS1–4 clustered with the Kawasaki genotype (96%–96.5% identity). Genotype FJS was closely related to the Fuji genotype (96.5% identity), which was originally isolated from *Leptotrombidium fuji* mites in Japan (*12*). Genotypes SDM1 and SDM2 clustered in an independent clade, which had 70.4%–77.9% identity with reference strains (Figure).

**Figure:**
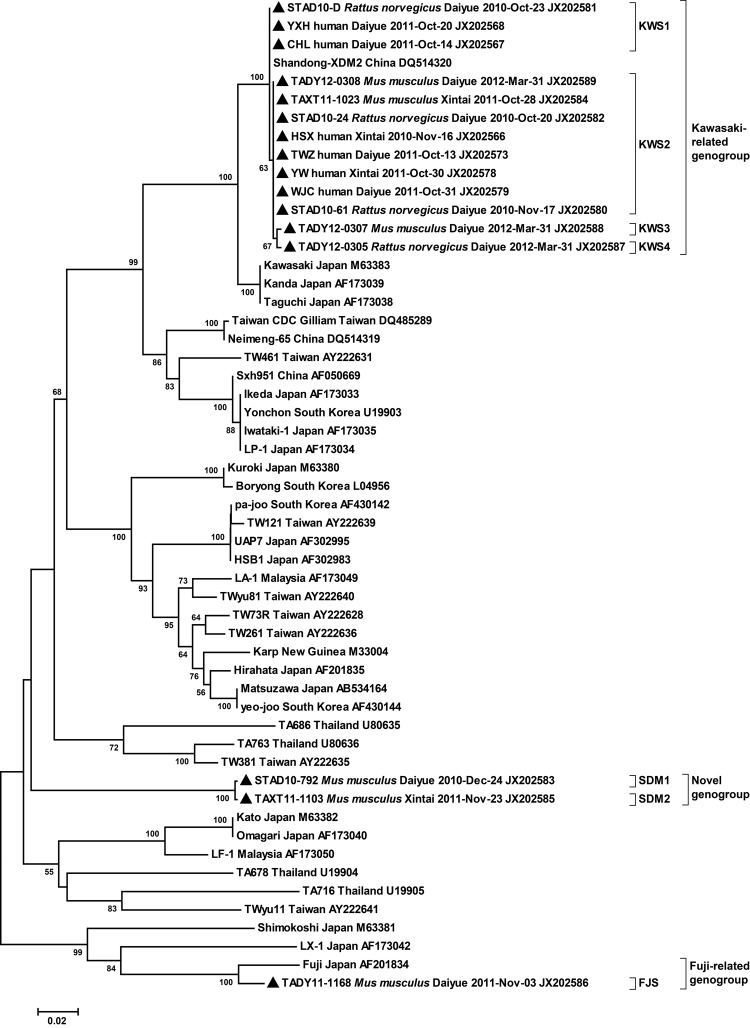
Phylogenetic relationships of *Orientia tsutsugamushi* detected in domestic rodents and human patients with scrub typhus in rural areas of Tai’an, Shandong Province, China, September 2010 through March 2012. Relationships were determined on the basis of the partial 56-kDa type-specific antigen gene of *O. tsutsugamushi* by the minimum-evolution method with the Kimura 2-parameter distance model. Bootstrap values >50% are shown at the branches. Location and GenBank accession numbers are indicated for each sequence. Sampling sources and sampling date are also indicated for sequences determined in the study. Solid triangle indicates sequences determined in this study. Scale bar indicates genetic distance.

Whole blood or eschar specimens were obtained from 19 human patients, among whom 6 (31.6%) were infected with *O. tsutsugamushi* according to seminested PCR. Two genotypes (KWS1 and KWS2), which were identified in domestic rodents and belonged to Kawasaki-related genogroups, were also found in the patients (Figure).

## Conclusions

Our study demonstrated the prevalence of 7 genotypes of *O. tsutsugamushi* in the new epidemic area in Tai’an, northern China. Genetic diversity of *O. tsutsugamushi* was observed within rodent species. Given that KWS1 and KWS2 identified in domestic rodents could cause human infection, prevalence of *O. tsutsugamushi* infection in domestic rodents might be a public health concern. Natural infection with the KWS2 genotype of *O. tsutsugamushi* in rodents was found during March in Tai’an. Periodic molecular surveillance of *O. tsutsugamushi* in local areas might help predict changes in the epidemic features of scrub typhus. Physicians should not arbitrarily exclude a diagnosis of scrub typhus for patients with fever of unknown origin during nonepidemic seasons.

The Kawasaki and Fuji strains of *O. tsutsugamushi* were considered to be of low virulence (*12*,*13*). Cross-reactivities of the Kawasaki strain with 1–18 anti-Gilliam monoclonal antibodies and of the Fuji strain with 5D-3 anti-Kato monoclonal antibodies were observed (*12*). The 56-kDa TSA is responsible for adhesion to and invasion of the host cells, and its variable domains might serve as epitopes (*5*,*14*). Although the identity of the KWS1–4 and FJS genotypes and their prototypes was >96%, changes in virulence and antigenicity might occur because of minor variation. Among the scrub typhus patients from Xintai and Daiyue, Kawasaki-related genotypes were predominant. Antigenic types of these genotypes remain to be determined. Those that are antigenically distinguished from the commonly used antigens should be included in the antigen panel of serologic assays in the local area, which would prompt the diagnosis and prevention of scrub typhus for local residents and travelers.

Identity of SDM1 and SDM2 with the reference strains was <77.9%, and they formed a distinct cluster, indicating that they might represent novel genotypes. Infection of scrub typhus patients with SDM1, SDM2, and Fuji-related genotypes has not been reported. Investigation of host ranges, invasiveness, and virulence of newly defined genotypes of *O. tsutsugamushi* in local areas will help determine their pathogenic potential for humans.

Expansion of genetic diversity of *O. tsutsugamushi* was expected to occur in maintenance hosts (*15*). We are currently screening *O. tsutsugamushi* in chigger mites collected from the captured rodents to evaluate the role of domestic rodents in the transmission of scrub typhus to humans. Assiduous surveillance of genetic variations of *O. tsutsugamushi* in hosts and identification of their pathogenic potentials is essential for the improvement of diagnostic capacity, vaccine development, and assessment of epidemiologic role.
